# Microwave-assisted synthesis of self-assembled C-doped-ZnO/g-C_3_N_4_ heterojunction catalysts for effective photodegradation of ofloxacin antibiotic†

**DOI:** 10.1039/d5na00060b

**Published:** 2025-03-31

**Authors:** Thi Viet Ha Luu, Ngoc Nhiem Dao, Van Vinh Nguyen, Quang Bac Nguyen, Thi Ha Chi Nguyen, Ngoc Chuc Pham, Ngoc Hoanh Dao, Trung Kien Nguyen

**Affiliations:** a Faculty of Chemical Engineering, Industrial University of Ho Chi Minh City 12 Nguyen Van Bao Street Ho Chi Minh City 700000 Vietnam; b Institute of Materials Science, Vietnam Academy of Science and Technology 18 Hoang Quoc Viet Street, Cau Giay Hanoi 100000 Vietnam nhiemdn@ims.vast.ac.vn nguyentrungkien1009@gmail.com; c Graduate University of Science and Technology, Vietnam Academy of Science and Technology 18 Hoang Quoc Viet Street, Cau Giay Hanoi 100000 Vietnam; d Joint Vietnam-Russia Tropical Science and Technology Research Center 63 Nguyen Van Huyen Street, Cau Giay Hanoi 100000 Vietnam; e Faculty of Mechanical Technology, School of Mechanical and Automotive Engineering, Hanoi University of Industry 298 Cau Dien, Bac Tu Liem Hanoi 100000 Vietnam

## Abstract

In this study, carbon-doped zinc oxide (CZ45) prepared using the microwave-assisted solvothermal method was electrostatically assembled with graphitic carbon nitride (GCN) to obtain CZ45/GCN (CZCN) heterojunction photocatalysts. The obtained composites showed average sizes in the range of 19.12–20.51 nm with the disintegration of petal-like stacked GCN sheets. A significant decrease in the bandgap (*E*_g_) from 3.12 eV in CZ45 to 2.67–2.81 eV in the CZCN composites and the photoluminescence (PL) spectra indicated an enhanced charge carrier separation suitable for the catalytic application under visible light irradiation. The CZCN11 composite (*E*_g_ = 2.81 eV) with a CZ45 : GCN weight ratio of 1 : 1 demonstrated outstanding photocatalytic performance in the degradation of ofloxacin (OFL) antibiotics compared to the other prepared CZCN composites as well as GCN and CZ45. The optimal parameters for OFL photodegradation by CZCN11 were determined; the CZCN11 dosage, OFL initial concentration, and pH range were found to be 1.01 g L^−1^, 20 ppm, and 7.0–8.0, respectively. Under these conditions, about 96% of the initial amount of OFL was decomposed at an apparent rate of 0.0173 min^−1^ in 180 min. A reusability test indicated the excellent durability and recyclability of CZCN11 in OFL photodegradation since the degradation efficiency was reduced only by about 1% after five successive runs without any alteration in the original structure of the composite. Furthermore, the active-charge-trapping experiments displayed the crucial role of superoxide (˙O_2_^−^) radicals in OFL photodegradation by the CZCN composites.

## Abstract 2

C-ZnO/g-C_3_N_4_ (*E*_g_ = 2.81 eV) self-assembled from as-prepared C-ZnO and g-C_3_N_4_ effectively photodegrades ofloxacin, a fluoroquinolone antibiotic, under visible-light irradiation in aqueous solutions.

## Graphical abstract

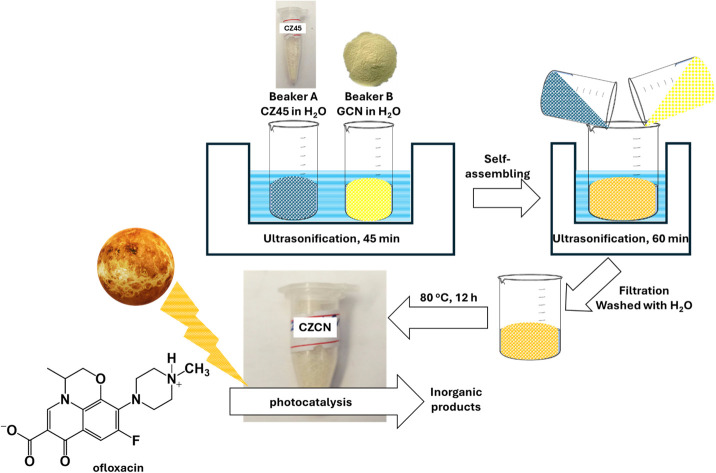

## 1. Introduction

Since the discovery of penicillin in 1928, antibiotics have become one of the most popular and effective medications for the treatment of numerous infectious diseases in humans, agricultural crops and animal husbandry.^1–4^ In addition, the production and consumption of antibiotics have increased tremendously because of the rapid growth of the human population worldwide and the huge advancements in technology and related fields.^5–7^ Despite the advantage of the remarkable reduction in the mortality rate and mortality due to common infectious diseases, the overproduction and overuse of antibiotics have contrarily led to the massive discharge of antibiotic residues into various aquatic environments, such as rivers, lakes, and groundwater, making them a group of emerging pollutants.^8–10^ The occurrence of antibiotics and their degradation/metabolite products in aquatic environments creates significant ecological risks like the growth inhibition of algae and useful bacteria or the development of antibiotic resistance in these species.^11–13^ Hence, the development of efficient strategies for the elimination of free antibiotics and their residues from the ecosystem is imperative.

Photocatalysis, an advanced oxidation process (AOP), has attracted significant attention in wastewater treatment.^14^ In photocatalysis, the photocatalytic materials absorb sufficient light energy to generate electrons (e^−^) and holes (h^+^) at the conduction band (CB) and valence band (VB), respectively. The photogenerated e^−^ and h^+^ then interact with the dissolved oxygen molecules and water to create superoxide (˙O_2_^−^) and hydroxyl (˙OH) radicals, which attack the organic pollutants and mineralize them into final non-toxic inorganic products like CO_2_ or H_2_O. This technique has advantages such as high degradation rate, non-selectivity, mild degradation conditions, environmental friendliness, and low cost.^15,16^ However, photocatalysis also faces some disadvantages. Even the most commonly used semiconductors, such as TiO_2_ and ZnO, exhibit a large bandgap of ≥ 3.2 eV and can only be activated by UV irradiation, which accounts for less than 5% of the solar light, hence limiting their wide practical application.^17,18^ Furthermore, related studies have indicated that a high recombination rate of e^−^/h^+^ hampers the remediation of organic contaminants. Several methods have been developed to promote the photocatalytic activity of materials and surpass their limitations to improve their photocatalytic capability in practical applications. The proposed strategies include altering the morphology and crystal structure, doping with specific elements, depositing plasmonic nanoparticles, establishing heterojunctions, utilizing a photosensitizer, and immobilizing the semiconductor on support carriers.^19,20^

ZnO and GCN semiconductors have been used as photocatalysts in various applications. ZnO is considered an alternative to TiO_2_, which has been employed since the dawn of photocatalysis, as it offers the advantages of high quantum efficiency, exciton binding energy and conductivity, distinct oxidation activity and chemical stability, potential heterojunction formation capability, good antifouling and antibacterial properties, low cost, and environmental friendliness.^21–23^ However, as mentioned above, the practical applications of ZnO are restricted because of its large bandgap (≥3.2 eV) and high rate of photoinduced charge recombination.^24^ On the other hand, GCN is a non-toxic metal-free polymer semiconductor with a bandgap of about 2.7 eV and is active under visible-light irradiation.^25–27^ GCN demonstrates strong reduction ability, simple preparation from common low-cost precursors (urea, thiourea, melamine, *etc.*), ease of combination with other semiconductors for the fabrication of heterojunction photocatalysts, non-toxicity, and relatively high thermal and chemical stability.^26–28^ Nonetheless, GCN photocatalysts also face several challenges, including the fast recombination of e^−^/h^+^ pairs, low specific surface area with a low density of active catalytic centers, moderate oxidative activity, and poor charge carrier mobility.^26–28^ Therefore, both ZnO and GCN require appropriate improvements to broaden their use in practical wastewater treatment plants.

In previous studies, our groups have successfully promoted the photocatalytic performance of ZnO by introducing C, Ce, and Ta dopants.^29–31^ Hence, in this study, we have attempted to elevate the photocatalytic activity by establishing heterojunction photocatalysts composed of C-doped-ZnO (CZ45) and GCN. CZ45 was prepared using a simple microwave-assisted solvothermal process, whereas GCN was synthesized by urea pyrolysis. Subsequently, based on electrostatic attraction, the as-prepared components were self-assembled at different weight ratios in a neutral aqueous solution to form the CZ45/GCN composites (CZCN).^32^ Subsequently, the physical and chemical characteristics of the obtained materials were thoroughly examined before they were employed as photocatalysts for the degradation of ofloxacin, which is a typical fluoroquinolone antibiotic, under visible-light irradiation. Finally, a degradation mechanism is also proposed and discussed.

## 2. Experimental methods

### 2.1. Chemicals

Zinc nitrate (Zn(NO_3_)_2_·6H_2_O, ≥99%), polyvinyl alcohol (PVA, MW = 146 000–186 000 g mol^−1^, ≥99%), urea (≥99%), nitric acid (HNO_3_, 70%), sodium hydroxide (NaOH), hydrochloric acid (HCl, 36%), ethanol (EtOH), silver nitrate (AgNO_3_), ethylenediamine tetraacetic acid (EDTA), benzoquinone (BQ), and isopropyl alcohol (IPA) were purchased from Sigma-Aldrich. Ofloxacin (≥98%) was domestically provided by the National Institute of Drug Quality Control of Vietnam. All chemicals and reagents were directly used as received from the provider without any further purification.

### 2.2. Photocatalyst preparation

#### Preparation of GCN

5 g of urea was crushed, placed in a ceramic crucible, and calcined for 2 h at 550 °C. After calcination, 15 mL of 0.1 mol L^−1^ HNO_3_ was added dropwise to the mixture and stirred using a magnetic stirrer for 15 min. Then, the solid was separated from the mixture by filtration and washed with deionized water until a neutral pH (pH 7.0) was reached. Finally, the solid product was dried at 80 °C for 12 h to obtain pale yellow GCN.

#### Preparation of C-ZnO

1.8 g of Zn(NO_3_)_3_·6H_2_O was dissolved in Beaker 1 containing 50 mL deionized water and 50 mL EtOH. Meanwhile, 0.09 g of PVA was completely dissolved in Beaker 2 containing 50 mL deionized water at 60 °C and stirred using a magnetic stirrer for 45 min. The solution in Beaker 1 was then transferred to Beaker 2 and stirred for 30 min at ambient temperature. After that, 100 mL of 0.6 mol L^−1^ NaOH was added to the mixture and stirred for 45 min for the precursors to completely dissolve. After that, the mixture was transferred to a 500 mL round flask and exposed to microwave (800 W, 2450 MHz) for 45 min. The microwave oven was attached to an external refluxing system. Consequently, the reacted solution was cooled down before the resulting solid was filtered and washed several times with deionized water until it was neutralized (pH 7.0). The solid was then heat-treated at 100 °C for 4 h before calcination at 500 °C for 2 h in an oven to obtain a final white powder, which is denoted as CZ45.

#### Preparation of CZ45/GCN (CZCN) composites

The CZCN composites were prepared from the as-prepared GCN and CZ45 at various CZ45/GCN mass ratios (w/w), including 6 : 1 (CZCN61), 1 : 1 (CZCN11), and 1 : 6 (CZCN16), according to a previously described method.^32^ For the preparation of CZCN11, 0.1 g and 0.1 g of CZ45 and GCN, were added to Beakers A and B containing 20 mL deionized water each and ultrasonicated for 45 min. After that, the mixtures in Beakers A and B were poured into another 100 mL beaker and ultrasonicated for 60 min. Subsequently, the resulting solid was filtered, washed with deionized water, and heat treated at 80 °C for 10 h to obtain the final powder. The same procedure was employed for CZCN61 and CZCN16 using corresponding weights of CZ45 and GCN.

### 2.3. Material characterization

The phase and crystal structure of the samples were examined using X-ray diffraction (XRD) on a Bruker D8 Advance X-ray diffractometer (Germany) with a Cu Kα radiation source. The Scherrer equation was employed to calculate the average crystal size of samples from the obtained XRD results (eqn (1)).1
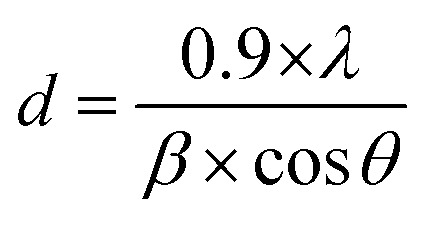
where *d*, *λ*, and *β* are the average crystal size, the wavelength of the X-ray radiation, and full-width at half maximum radian, respectively.

Then, the microstructure and morphology of the obtained materials were assessed using scanning electron microscopy (SEM) on a Hitachi S-4800 instrument (Japan) operated at a voltage of 10 kV and high-resolution transmission electron microscopy (TEM, HR-TEM) on an S-4800 NIHE model (Japan). The elemental composition of the CZCN samples was assessed by employing energy-dispersive X-ray spectroscopy (EDX) in conjunction with the SEM system. The oxidation states of the elements found in CZCN were determined by X-ray photoelectron spectroscopy (XPS) on a PHI Quantera SXM apparatus (ULVAC-PHI, Japan) using an Al Kα radiation (1486.6 eV, 10 kV, 20 mA). The binding energy (BE) of the XPS spectra was calibrated using the Au standard powder with 4f_7/2_ peaks at 84.0 eV. The optical properties and *E*_g_ of the obtained materials were analyzed by UV-vis diffuse reflectance spectroscopy using a JASCO V500 UV-vis spectrometer (Japan). The photoluminescence spectra were acquired at a 355 nm excitation wavelength on a Horiba HQ iHR550 (Japan). The isoelectric points (pHz) of CZ45 and GCN were determined by the drift method,^33^ and the results are reported in Fig. S1.†

### 2.4. OFL photodegradation

OFL photodegradation was carried out using the CZCN photocatalysts on a photoreactor from ACE Glass Inc. (U.S.A). The irradiation source was a 450 W low-pressure mercury vapor lamp positioned inside a 500 mL Pyrex immersion well. The light intensity on the exterior arc surface of the lamp and the well was 1.04 and 0.37 W cm^−2^, respectively. An external water stream was installed around the photoreactor to control its temperature. A 250 mL mixture containing OFL and the photocatalyst was continuously stirred for 60 min in darkness using a magnetic stirrer to reach the OFL adsorption/desorption equilibrium on the CZCN catalyst before the light source was switched on. The photocatalysts (CZ45, GCN, CZCN61, CZCN11, and CZCN16), pH of the mixtures (4.0–10.0), catalyst dosage (0.52, 0.71, 1.01, and 1.25 g L^−1^), and initial OFL concentration (15, 20, 30, and 40 ppm) were systematically changed to examine the influence of these parameters on OFL photodegradation. The reactive-species-trapping experiments were conducted by adding IPA, EDTA, AgNO_3_, and BQ as scavengers of hydroxyl radicals, generated holes, electrons, and superoxide radicals, respectively. After a certain time, 1 mL of the working mixture was collected. The catalyst residues were isolated from the mixture by centrifugation, and the absorbance of the aliquot was analyzed on a UV-vis spectrometer at the wavelength of 288 nm. For the reusability test, after calculating the remaining amount of OFL in the final solution, an appropriate amount of OFL was added to the photoreactor, and the pH was re-adjusted using HCl and KOH to reach the initial reaction conditions. The mixture was then subjected to the same procedure, starting with the stirring in the darkness. The reusability was tested for five consecutive cycles. After the fifth cycle, the catalyst was collected, isolated from the solution by filtration and centrifugation, washed several times with deionized water, and dried at 80 °C for 12 h for subsequent structural assessment using SEM and XRD techniques. The mineralization of OFL during the first photodegradation cycle under optimal conditions was assessed based on the reduction in total organic carbon (TOC) using an OI Analytical Aurora 1030C TOC Analyzer.

The degradation efficiency (DE) of OFL at time *t* was determined using the following equation (eqn (2)).2
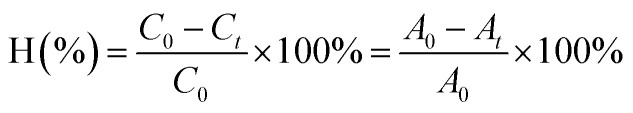
where *A*_0_ and *A*_*t*_ correspond to the absorbance values of AMX at 0 and *t* min, and *C*_0_ and *C*_*t*_ represent the concentrations of AMX at 0 and *t* min, respectively.

The apparent degradation rate *k*_app_ for the pseudo-first-order kinetics model was evaluated as follows (eqn (3)).3
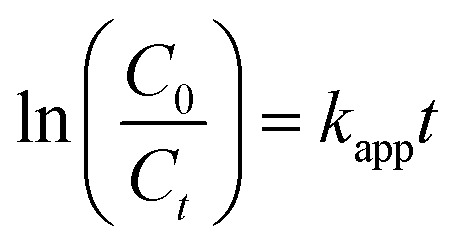


## 3. Results and discussion

### 3.1. Material characterization

**Fig. 1:**
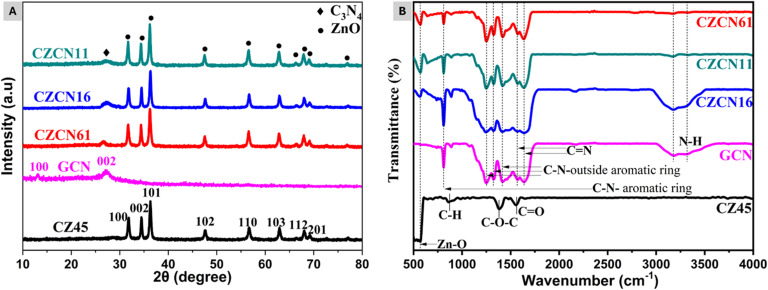
(A) Diffractograms and (B) FTIR spectra of the synthesized CZ45, GCN, and CZCN samples.

Fig. 1A demonstrates the diffraction patterns of prepared CZ45, GCN, and CZCN materials containing different weight ratios of CZ45 : GCN. GCN displayed a weak peak and a broad peak centered at diffraction angles (2*θ*) of 13.1° and 27.1°, corresponding to the (100) and (002) planes (JCPDS standard No. 87-1526),^34^ respectively. The observed peaks are attributed to the in-plane structural packing motif of GCN and graphitic stacking in C_3_N_4_.^35^ Meanwhile, CZ45 illustrated a diffraction pattern that could be associated with the structure of hexagonal wurtzite ZnO crystals (JCPDS standard No. 036-1451).^36^ Its diffraction peaks could be ascribed to (100), (002), (101), (102), (110), (103), (112), and (201) planes. On the other hand, the curves of all prepared CZCN samples demonstrated the characteristic peaks of both GCN and CZ45 but with differences in the peak intensities. As the CZ45 : GCN weight ratio in the CZCN samples increased, the peak intensity of the (002) plane of the GCN component decreased and almost disappeared, whereas those of the CZ45 component showed a slight increase. Particularly, the characteristic peak of the (100) plane of GCN was absent in all CZCN curves. Both these changes suggest the possible formation of linkages between the CZ45 and GCN components in the composite materials. According to the XRD results, the average crystalline sizes of CZ45, CZCN16, CZCN11, and CZCN61 calculated using the Scherrer equation were 21.13 nm, 19.12 nm, 20.51 nm, and 19.91 nm, respectively.

FTIR spectral analysis was then employed to verify the possible bonds and functional groups present in the prepared samples (Fig. 1B). In the curve of CZ45, the strong absorption peak at 567 cm^−1^ was attributed to the Zn–O bond vibration, whereas the peaks in the range of 800–1700 cm^−1^ were assigned to the C–H, C–O–C, or C=O bonds arising from the C-dopants or solvent residues.^37,38^ In the spectrum of GCN, a broad band with doublet peaks in the range of 3000–3600 cm^−1^ was observed due to the stretching vibration of hydrogen bonds, including N–H (3177 cm^−1^) and O–H (3319 cm^−1^) bonds.^35,39,40^ Besides, the typical peaks in the range of 1000–1800 cm^−1^ could be ascribed to the stretching vibration of the C–N bonds in the aromatic rings of GCN.^35,39,40^ Moreover, an intense absorption at 810 cm^−1^ revealed the strain vibration of the triazine rings.^35,39,40^ Most of the characteristic bands of the CZ45 and GCN components were also present in the corresponding CZCN curves. However, their peak intensities varied based on the CZ45 : GCN ratio in the composites. A higher proportion of the CZ45 component resulted in high-intensity peaks at about 570 cm^−1^ due to the Zn–O bonds, whereas the intensities of the GCN-associated bands arising from the triazine rings, N–H bonds, and aromatics rings were significantly reduced.^40^

**Fig. 2:**
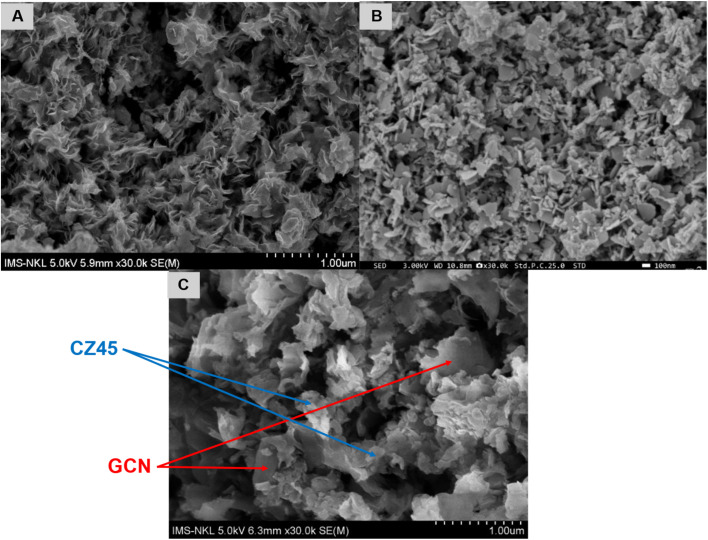
SEM images of (A) GCN, (B) CZ45, and (C) CZCN11 samples.

The microstructure of the prepared samples was subsequently examined using the SEM technique. Fig. 2A exhibits the petal-like arrangement of the GCN layers, whereas CZ45 displayed the formation of fragmented sheets with partial aggregation of particles (Fig. 2B). On the other hand, when the CZCN composite was assembled, the attachment of CZ45 fragments on the GCN sheets was evident (Fig. 2C). In the CZCN composites, larger sheets were formed with lesser aggregation of CZ45 particles along with the disintegration of the GCN petal stacks.

**Fig. 3:**
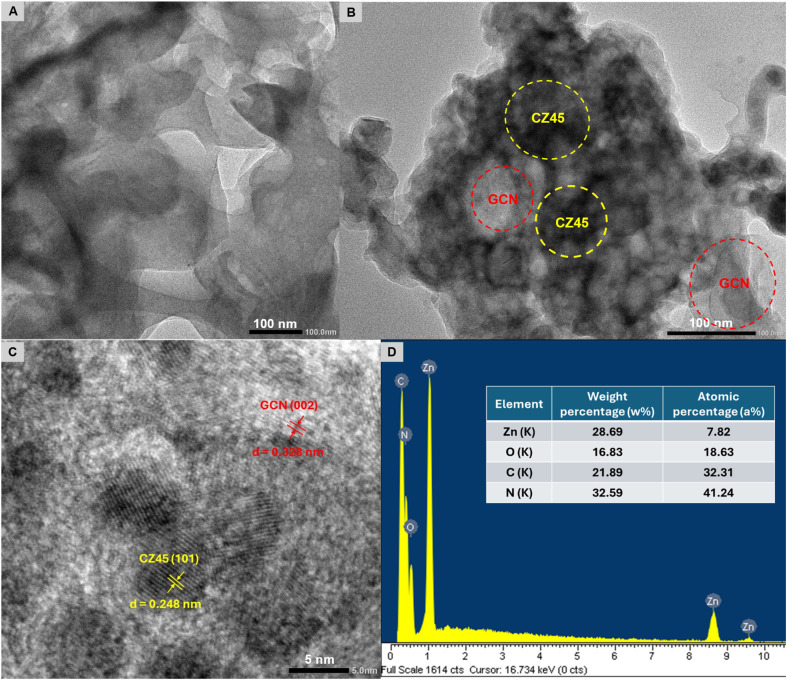
TEM images of (A) GCN and (B) CZCN11 samples. (C) HR-TEM image and (D) EDX spectra of the CZCN11 sample (inset table: elemental composition of the CZCN11 sample).

The captured TEM image of GCN also exhibited the layer-stacked morphology of the prepared materials as grey regions (Fig. 3A). Meanwhile, the black regions in the TEM image of the CZCN11 sample indicated the CZ45 particles attached to GCN layers as the composites were formed (Fig. 3B). In addition, an HR-TEM image of the CZCN11 sample also revealed the co-existence of CZ45 and GCN phases in CZCN (Fig. 3C). The lattice spacing *d* values of 0.248 nm and 0.328 nm were associated with the (101) plane of CZ45 and the (002) interlayer reflection of a graphite-like structure of GCN, respectively. On the other hand, Fig. 3D shows the EDX spectra of CZCN11 with the elemental composition in the inset table, confirming the presence of Zn, O, C, and N and the absence of impurities in the prepared sample. Assuming the weight percentage of the C-dopant in CZ45 was 10 wt% of ZnO, the obtained elemental composition also indicates approximately the same weight ratio of CZ45 : GCN in CZCN11 as that of the precursors, suggesting the preservation of elements throughout the preparation step.

**Fig. 4:**
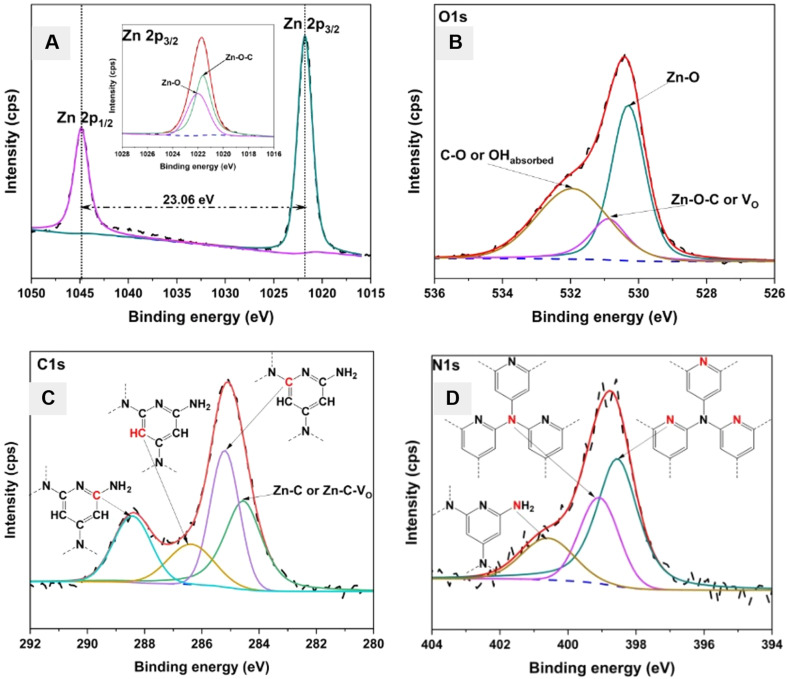
High-resolution XPS spectra of the (A) Zn 2p (inset figure: the deconvolution of Zn 2p_3/2_ peak), (B) O 1s, (C) C 1s, and (D) N 1s core levels of the CZCN11 composite (black dashed line: raw data, blue dashed line: baseline, red line: fitting line, other lines: deconvoluted lines).

An XPS survey of the CZCN11 sample was conducted to examine the chemical composition and valence states of the included elements (Fig. S2†). The full scan with the corresponding Zn 2p, O 1s, C 1s, and N 1s core level spectra showed that the obtained composite contained zinc, oxygen, carbon, and nitrogen. Fig. 4A reveals a doublet at the binding energies (BE) of 1021.79 eV and 1044.85 eV associated with Zn 2p_3/2_ and Zn 2p_1/2_, respectively. These BE values and the difference between them (23.06 eV) suggest that Zn was in the Zn^2+^ state of ZnO according to the reference values.^41,42^ The inset figure in Fig. 4A shows the possible deconvolution of Zn 2p_3/2_ into two components, including Zn–O–C due to the C-dopant and the linkages formed between CZ45 and GCN, and Zn–O in ZnO.^42^ The O 1s XPS spectrum and its deconvoluted peaks are shown in Fig. 4B. A main peak denoting O^2−^ in the Zn–O bond of the ZnO wurtzite structure and a shoulder peak for the C–O or OH group of absorbed CO_2_ or water molecules onto the surface of the composite were observed at 530.30 eV, and 531.94 eV, respectively.^43^ Besides, a peak positioned at 530.89 eV indicating the Zn–O–C bonds or oxygen vacancies (*V*_O_) formed by the doping of C atoms or the formation of linkages between GCN and CZ45 were also seen, consistent with previous studies.^43,44^ The high-resolution C 1s spectra illustrate a doublet, which could be deconvoluted into four different peaks representing the characteristic bonds in CZ45 and GCN components of the CZCN composite (Fig. 4C). The peak centered at 284.55 eV was attributed to carbon atoms in the carbide form (Zn-C or Zn-C-V_O_), possibly created both by the substitution of O atoms with C dopants in the ZnO matrices and linkage formation between the CZ45 and GCN components.^45,46^ The signals at 285.20 eV and 286.38 eV were attributed to pure graphitic sites inside the carbon nitride matrix and the sp^2^-hybridized carbon atoms bound to nitrogen in the aromatic ring, respectively.^46^ The peak at the highest BE (288.42 eV) corresponds to the sp^2^-hybridized carbon in the aromatic ring attached to the NH_2_ group.^45,47^ The N 1s XPS spectrum is demonstrated in Fig. 4D. The three deconvoluted peaks of the N 1s core level spectrum were centered at 398.55 eV, 399.08 eV, and 400.59 eV. They were assigned to the sp^2^-hybridized nitrogen (C–N=C) in triazine rings, the tertiary nitrogen N–(C)_3_ group, and the amino group C–N–(H)_2_ attached to the aromatic ring, respectively.^45^ In addition, a comparison of the BE values of the Zn 2p, O 1s, C 1s, and N 1s core levels of CZ45, GCN, and CZCN is presented in Fig. S3.† While the binding energies (BEs) of C 1s and N 1s of GCN showed positive shifts compared with those of CZCN, the BEs of Zn2p and O 1s of CZ45 displayed negatively shifts compared with those of CZCN. These findings suggest the occurrence of electron transfer between CZ45 and GCN when the CZCN composites were self-assembled, as described in a previous study by Nie *et al.*^32^ CZ45 acts as the electron acceptor, whereas GCN plays the role of an electron donor.

**Fig. 5:**
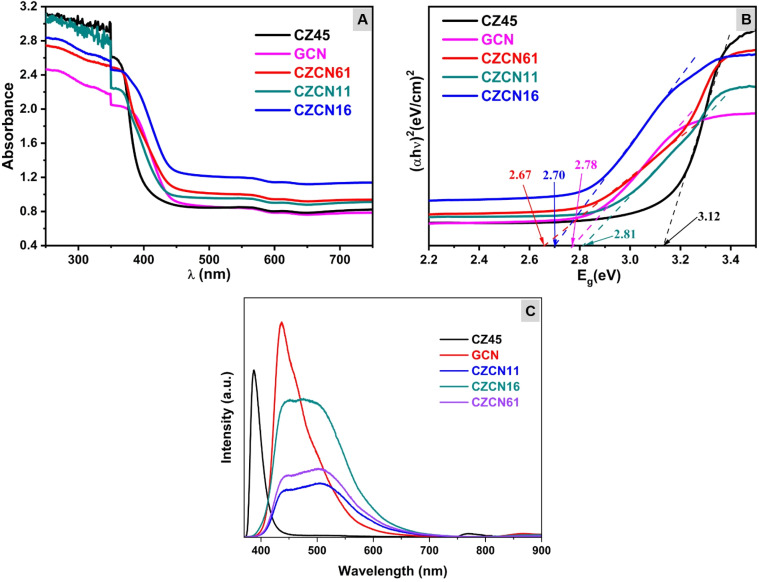
(A) UV-vis spectra, (B) derived Tauc plots, and (C) PL spectra of the prepared samples.

**Fig. 6:**
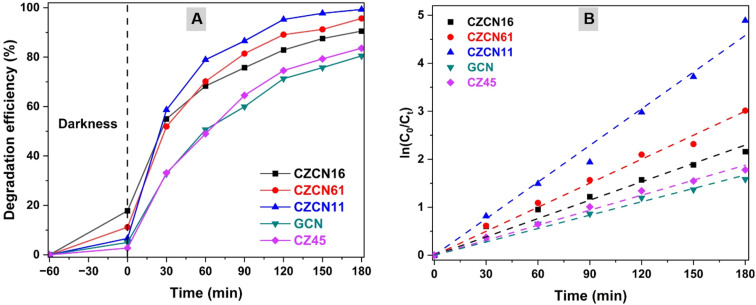
(A) Efficiency and (B) fitted first-order kinetic model for the photodegradation of OFL by the prepared materials.

The optical performance of all prepared composites was first investigated using UV-vis DRS. While CZ45 displayed a sharp UV absorption peak (*λ* < 400 nm), GCN and all prepared composites exhibited a remarkable redshift in the absorption edge to the visible-light region (*λ* > 400 nm) (Fig. 5A). According to the Tauc method,^46^ a plot was drawn, and the bandgap energies (*E*_g_) of CZ45, GCN, CZCN61, CZCN11, and CZCN16 were calculated to be 3.12 eV, 2.78 eV, 2.67 eV, 2.81 eV, and 2.70 eV, respectively (Fig. 5B). These results suggest that the obtained composites could harvest solar energy more effectively to initiate photocatalysis than ZnO (*E*_g_ ∼ 3.2 eV^48^) and CZ45. The photoluminescence (PL) of CZCN (CZCN11), CZ45, and GCN was then examined at room temperature using an excitation wavelength of 325 nm. Fig. 5C illustrates sharp emission peaks centered at 387 nm and 435 nm for the CZ45 and GCN samples, respectively. On the other hand, the composites made of CZ45 and GCN demonstrated broadened PL emission bands, with the maximum intensity in the range of 450–500 nm. However, compared with both CZ45 and GCN samples, the PL emission of CZCN exhibited lower intensities, especially those of CZCN11 and CZCN61. These results not only confirm the formation of a heterojunction structure between CZ45 and GCN but also suggest restriction of electron–hole recombination in the obtained CZCN composites^49^ (Fig. 6).

### 3.2. OFL photodegradation

First, the photocatalytic activity of all prepared materials and the effect of the CZ45 : GCN mass ratio in the composites on OFL degradation were evaluated. Under darkness, the OFL removal efficiencies of all materials were limited. When exposed to a light source, GCN and CZ45 displayed analogous degradation behavior, and the corresponding DE values reached 80.48% and 83.59% in 180 min. Meanwhile, the synthesized composites exhibited significantly enhanced OFL degradation, with the DEs surpassing 90% in the same irradiation time. CZCN11 showed the highest DE in the examined time as most OFL was photocatalytically degraded (99.29%), while CZCN16 and CZCN61 could also remove 90.51% and 95.6% of the initial OFL amount. Then, the kinetic behavior of the prepared materials was also evaluated based on the dependence of ln(*C*_0_/*C*_*t*_) on the reaction time. The linear fitting curves of all the materials were obtained, and the regression coefficient *R*^2^ values were close to 1, suggesting that OFL photodegradation catalyzed by the synthesized materials fitted well with pseudo-first-order kinetics. The calculated *k*_app_ increased in the order of GCN (0.0093 min^−1^) < CZ45(0.0104 min^−1^) < CZCN16(0.0128 min^−1^) < CZCN61 (0.0167 min^−1^) < CZCN11 (0.0254 min^−1^). In addition, the increase in the CZ45 : GCN ratio in the composite from 1 : 6 to 1 : 1 accelerated the photodegradation process, whereas a further increase to 6 : 1 slowed the reaction down, but it was still faster than those observed with pure GCN and CZ45. According to the UV-vis and PL spectra, the restricted photocatalytic OFL degradation activity of CZ45 and GCN might have resulted from fast charge recombination or harvesting insufficient solar energy due to the large *E*_g_ (CZ45). Since the heterojunctions in the CZCN catalysts have narrower bandgaps (2.67–2.81 eV) that enhance solar energy absorption and limit electron/hole recombination, the photodegradation process is more effective. Moreover, an excess amount of either component (CZ45 or GCN) in the composite also caused a negative impact (less energy harvesting or creating more recombination sites) on the photocatalytic activity. Consequently, CZCN11 was used in further OFL degradation studies to evaluate the influence of some crucial reaction conditions.

**Fig. 7:**
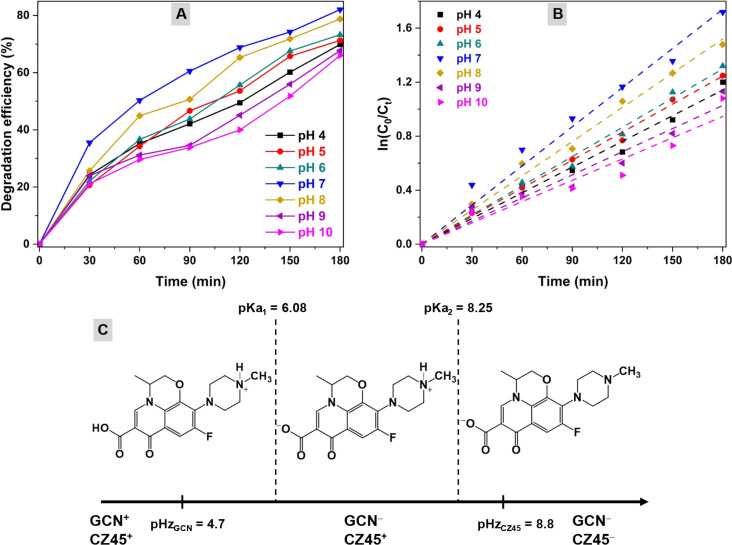
Influence of the pH of solution on the (A) OFL degradation efficiency and (B) degradation rate of the OFL photodegradation using the CZCN11 photocatalyst. (C) Ionic form of OFL and surface charges of the photocatalyst at different pH values.

The influence of pH on the photocatalytic OFL degradation performance was then evaluated. OFL photodegradation on CZCN11 was evaluated in a series of solutions in the pH range of 4–10 at a fixed OFL concentration of 40 ppm and catalyst dosage of 1.0 g L^−1^. The results reported in Fig. 7A show the increase in DE values in the following order: pH 10 (66.03%) < pH 9 (67.73%) < pH 4 (69.88%) < pH 5 (71.31%) < pH 6 (73.27%) < pH 8 (77.21%) < pH 7 (82.07%). An ascending trend of the degradation rate *k*_app_ was also observed, with the lowest and highest *k*_app_ values of 0.0053 min^−1^ and 0.0097 min^−1^, respectively (Fig. 7B). Since photodegradation commonly occurs on the surface of the catalyst, to explain the trend of the DE and *k*_app_ values in the tested pH range, the ionic forms of OFL molecules and surface charges of the GCN and CZ45 components in the CZCN composites are illustrated in Fig. 7C. According to the isoelectric point (pHz) and acidic dissociation constant (p*K*_a_), at low pH (pH 4), both OFL and the active sites on CZCN are positively charged. Consequently, CZCN and the OFL^+^ ions display electrostatic repulsion, which results in weak interactions. A similar behavior was observed when the pH was increased above pHz_CZ45_. The formation of OFL^−^ ions and the negative charges of both GCN and CZ45 sites also lead to electrostatic repulsion between the contaminant and the photocatalyst and limit the adsorption of OFL^−^ on CZCN during photocatalysis. In addition, as the pH increases to a more alkaline state, CZ45 may be dissolved partially, directly affecting the photodegradation efficiency. On the other hand, when the pH value is between pHz_GCN_ and pHz_CZ45_, the OFL ions (OFL^+^, OFL^±^, and OFL^−^) can be favorably adsorbed on the catalyst surface due to electrostatic attraction, resulting in more effective OFL photodegradation. Based on the obtained results, the subsequent OFL photodegradation experiments were conducted in the pH range of 7 and 8.

**Fig. 8:**
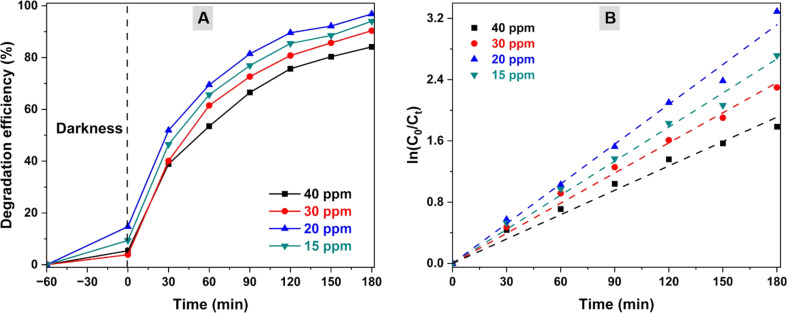
Influence of the initial OFL concentration on the (A) degradation efficiency and (B) degradation rate of OFL.

Fig. 8 demonstrates the OFL photodegradation process with various initial OFL concentrations in a pH7 solution containing 1.0 g L^−1^ of the CZCN11 catalyst. As the OFL concentration escalated from 15 ppm to 20 ppm, the DE increased from 94.00% to a peak at 96.82%, followed by a considerable decline to 90.34% and 84.13% with a subsequent increase in OFL concentration to 30 ppm and 40 ppm, respectively. The *k*_app_ values calculated from the curves fitted to pseudo-first-order kinetics also displayed an analogous trend. The *k*_app_ values of 15 ppm, 20 ppm, 30 ppm, and 40 ppm were 0.0148 min^−1^, 0.0173 min^−1^, 0.0131 min^−1^, and 0.0106 min^−1^, respectively. It is known that a fixed amount of photocatalyst allows a specific quantity of OFL contaminants to be adsorbed onto the surface or the active sites for degradation. An excessive amount of OFL would create an external barrier on the catalyst, resulting in limited contact between the incident light and the catalyst and consequently a reduction in photon energy absorbability. As a result, the initial OFL concentration of 20 ppm is considered the optimal OFL photodegradation.

**Fig. 9:**
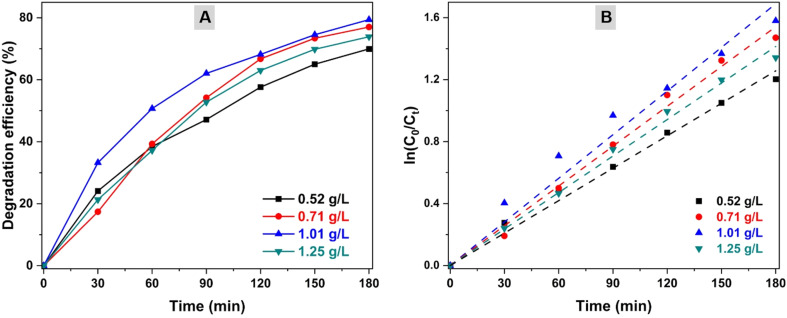
Influence of catalyst dosages on the (A) degradation efficiency and (B) degradation rate of OFL.

The catalyst dosage is another factor directly related to the photocatalytic pollutant degradation performance. The CZCN11 dosages were varied from 0.52 g L^−1^ to 1.25 g L^−1^ in a 40 ppm OFL solution at pH 7 (Fig. 9). In the CZCN dosage range of 0.52 g L^−1^ to 1.01g L^−1^, the DE significantly increased from 69.97% (0.52 g L^−1^) to 77.03% (0.71 g L^−1^) and reached the peak at 79.41% (1.01 g L^−1^). However, a decline in DE to 73.87% was seen with a further increase in CZCN amount to 1.25 g L^−1^. The *k*_app_ values corresponding to catalyst dosages of 0.52 g L^−1^, 0.71 g L^−1^, 1.01 g L^−1^, and 1.25 g L^−1^ calculated from the linear plot of ln(*C*_0_/*C*_*t*_) *vs.* reaction time were 0.0070 min^−1^, 0.0086 min^−1^, 0.0094 min^−1^, and 0.0079 min^−1^. An increase in catalyst quantity is known to enhance the number of active sites for catalysis, potentially generating active charges and radicals for the remediation of pollutants under irradiation. Hence, a considerable increase in the DE values of OFL photodegradation was observed. However, with an excess of catalyst in the solution, particle aggregation of the catalyst might occur, resulting not only in poor adsorbability of OFL molecules onto the CZCN surface but also lower photon absorbability of the catalyst to initiate photocatalysis. Therefore, the photocatalytic reaction becomes more sluggish and less effective.

**Fig. 10:**
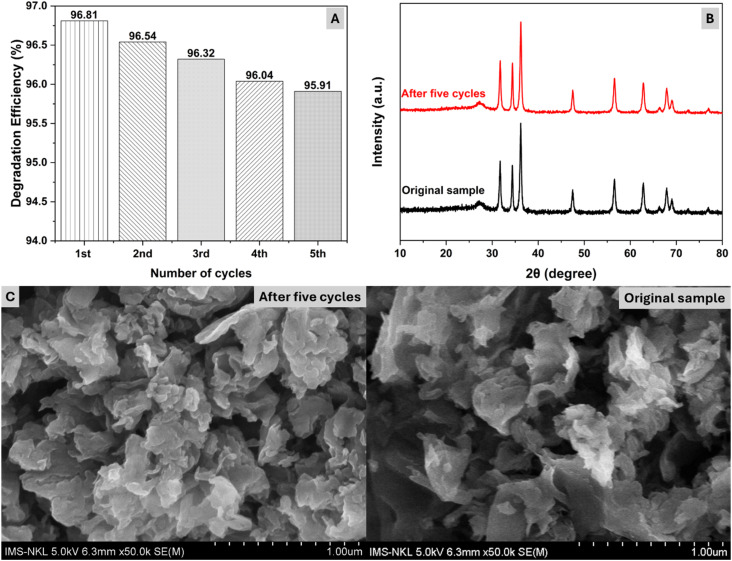
(A) Durability and recyclability test of the CZCN composite over 5 cycles of OFL photodegradation. (B) XRD patterns and (C) SEM images of the CZCN composite before and after the reusability test.

Finally, the durability and recyclability of the CZCN composite for OFL photodegradation were assessed by repeating the experiments several times (Fig. 10A). According to the above investigations on parameters related to OFL photodegradation, CZCN11, 1.0 g L^−1^, 20 ppm, and 7.0 were selected as the catalyst, catalyst dosage, initial OFL concentration and pH of the solution, respectively. According to the results shown in Fig. 10A, after five successive photodegradation cycles, the DE value displayed a change of less than 1%, from 96.81% in the first run to 95.91% in the fifth run. In addition, according to the XRD patterns and SEM images, the structure and morphology of CZCN11 also exhibited no noticeable changes. Therefore, CZCN11 demonstrates high stability and potential for OFL photodegradation in practical applications. Additionally, TOC removal was also measured in the first cycle of OFL photodegradation to assess mineralization to inorganic compounds. The initial TOC value of the ofloxacin solution (20 ppm) was recorded as 11.97 mg L^−1^, which chronically decreased by about 73.84% to 3.13 mg L^−1^ after 180 min of photodegradation using the CZCN photocatalyst. The obtained data suggest that even though the DE value approaches about 97%, the formation of a remarkable amount of intermediates still occurs during OFL photodegradation on CZCN materials.

### 3.3. Charge carrier trapping and the proposed degradation mechanism

**Fig. 11:**
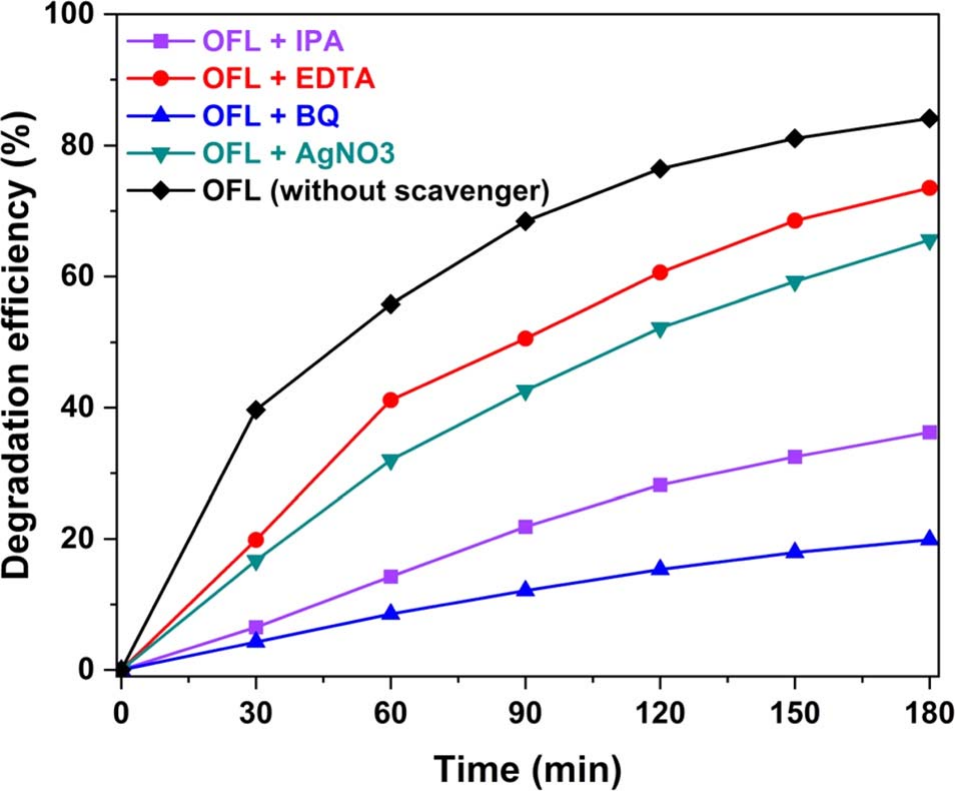
OFL photodegradation by CZCN11 with and without scavengers of the corresponding active charges.

To elucidate the role of the photogenerated active species in OFL degradation using CZCN, the corresponding scavengers of electrons, holes, hydroxyl radicals, and superoxide radicals, namely AgNO_3_, EDTA, IPA, and BQ, were added during the experiments (Fig. 11). In general, the results of OFL photodegradation with scavengers obviously showed varying degrees of decrease in the DE value, suggesting that all generated active charges participated in OFL photodegradation but differed in the level of contribution. While the DEs of OFL photodegradation with EDTA and AgNO_3_ displayed a slight decrease from 84.13% to 73.52% and 65.56%, respectively, the experiments in the presence of IPA and BQ exhibited a harsh drop in DE to 36.25% and 19.88%, respectively. These results suggest that the generated holes and electrons play minor roles, while ˙OH and especially ˙O_2_^−^ radicals play key roles in OFL degradation photocatalyzed by CZCN.

**Fig. 12:**
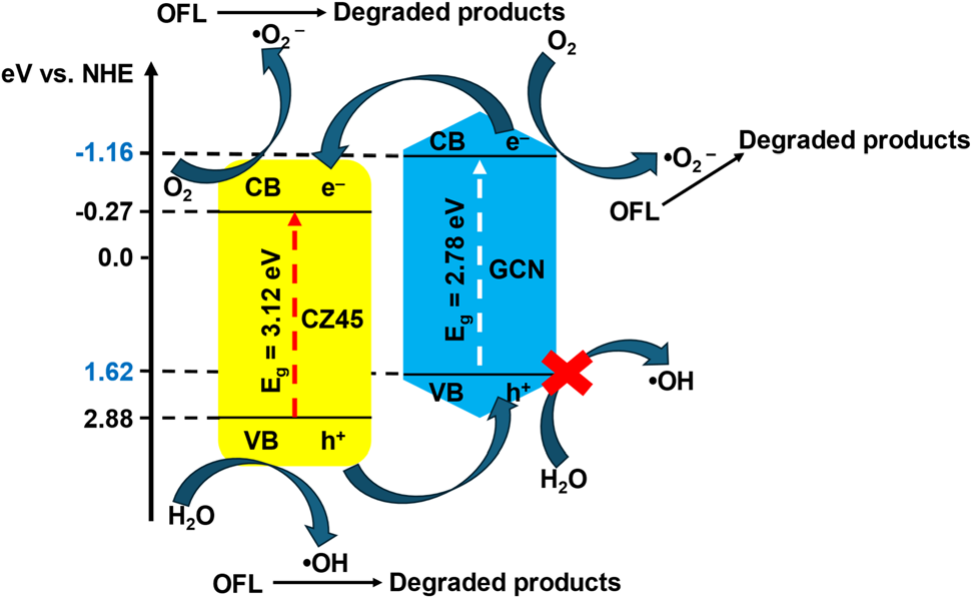
A plausible OFL photodegradation mechanism on the CZCN11 composite.

Based on active-charge trapping, a simple mechanism for OFL photodegradation using CZCN is proposed (Fig. 12). The positions of the CBs and VBs of CZ45 and GCN were determined according to the following equations (eqn (4) and (5)).^50,51^

4 *E*_VB_ = *χ* − *E*^e^ + 0.5*E*_g_

5 *E*_CB_ = *E*_VB_ − *E*_g_

where *E*^e^ is the free electron energy on the hydrogen electrode scale (4.5 eV), *E*_CB_ and *E*_VB_ are the energies of the CB and VB, respectively, and *χ* is the electronegativity of ZnO (5.79 eV) and GCN (4.73 eV) in the Mulliken scale. From the *E*_g_ values shown in Fig. 5, the *E*_CB_(CZ45), *E*_VB_(CZ45), *E*_CB_(GCN), and *E*_VB_(GCN) were calculated to be −0.27 eV, 2.85 eV, −1.16 eV, and 1.62 eV, respectively.

Upon exposure to light sources, when the electrons in the VBs absorb enough energy to be excited, they move to the CBs, resulting in the formation of active photogenerated electrons and holes. Because *E*_CB_(GCN) is more negative than *E*_CB_(CZ45), whereas *E*_VB_(CZ45) is more positive than *E*_VB_(GCN), it is likely that the excited electrons in the CB of GCN move to the CB of CZ45, while the holes generated in the VB of CZ45 migrate to the VB of GCN. Since both *E*_CB_(GCN) and *E*_CB_(CZ45) are more negative than the standard redox potential of ˙O_2_^−^/O_2_ (−0.046 V *vs.* NHE),^52,53^ the excited electrons react with dissolved O_2_ in the solution to create ˙O_2_^−^ radicals, which later degrade the OFL molecules. On the other hand, because *E*_VB_(GCN) is less positive than the standard redox potentials of OH^−^/˙OH (+1.99 V *vs.* NHE)^52,53^ and OH^−^/H_2_O (+2.8 eV. NHE),^54^ the holes h^+^ on the VB of GCN cannot react with H_2_O or OH^−^ to produce ˙OH. Hence, the ˙OH radicals are more likely to be formed in the VBs of the CZ45 sites for the remediation of OFL. The majority of holes in the VB of CZ45 serve as the sources of ˙OH rather than being transferred to the VB of GCN. Therefore, because of this approach, charge recombination in the CZCN composite might be restricted, while the electron transfer on the surface of the catalyst is simultaneously enhanced, leading to an improvement in photocatalytic activity compared with pure GCN and CZ45. The OFL photodegradation process on CZCN can be described by the following equations (eqn (6)–(11)).6
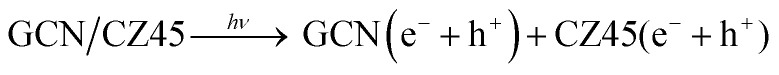

7 GCN (e_CB_^−^) → CZCN (e_CB_^−^)

8 e^−^ + O_2_ → ˙O_2_^−^

9 CZ45 (h_VB_^+^) + H_2_O → CZ45 + ˙OH + H^+^

10 CZ45 (h_VB_^+^) + OH^−^ → CZ45 + ˙OH

11 e^−^ + ˙O_2_^−^ + ˙OH + h^+^ + OFL → degraded products

## 4. Conclusion

This study reports the successful preparation of heterojunction photocatalysts using C-doped-ZnO (CZ45) and GCN at different weight ratios through a self-assembly process. CZ45 was synthesized *via* a simple microwave-assisted solvothermal method, while GCN was produced through urea pyrolysis. The synthesized composites exhibited average particle sizes in the range of 19.12–20.51 nm, close to the size of CZ45 (21.13 nm). Moreover, the disintegration of the petal-like stacked GCN sheets was also observed in the formed CZCN composites. Investigation using UV-vis DRS illustrated a notable alteration in *E*_g_ values from 3.12 eV in CZ45 to 2.67–2.81 eV in the CZCN powders, enabling visible-light absorption by the obtained composites. Additionally, the photoluminescence (PL) spectra signified an improvement in charge carrier separation in CZCN compared with CZ45. The CZCN11 composite (*E*_g_ = 2.81 eV) with a CZ45 : GCN weight ratio of 1 : 1 exhibited exceptional photocatalytic efficacy in the degradation of ofloxacin (OFL), a fluoroquinolone antibiotic, in comparison with the other synthesized CZCN composites, as well as the individual GCN and CZ45 components. The optimal conditions for OFL photodegradation using CZCN11 were established as follows: CZCN11 dose at 1.01 g L^−1^, initial OFL concentration at 20 ppm, and pH range between 7.0 and 8.0. Under these conditions, almost 96% of the initial OFL concentration was degraded at an apparent rate of 0.0173 min^−1^ in 180 minutes. A reusability test demonstrated the exceptional durability and recyclability of CZCN11 in OFL photodegradation, as the degradation efficiency declined only by around 1% after five consecutive uses, with no alteration to the original structure of the composite. The active-charge-trapping experiments demonstrated the most important role of superoxide (˙O_2_^−^) radicals in OFL photodegradation using the CZCN composites.

## Data availability

All data and materials generated or analyzed during this study are included in this published article and the ESI† file and are available from the corresponding author upon reasonable request.

## Author contributions

Thi Viet Ha Luu: methodology, conceptualization, investigation; Ngoc Nhiem Dao: supervision, validation, writing – review and editing; Van Vinh Nguyen: investigation, formal analysis; Quang Bac Nguyen: software, visualization; Thi Ha Chi Nguyen: investigation, formal analysis; Ngoc Chuc Pham: investigation, data curation; Ngoc Hoanh Dao: software, data curation; Trung Kien Nguyen: project administration, funding acquisition, writing – original draft.

## Conflicts of interest

The authors declare no conflict of interest.

## Supplementary Material

NA-OLF-D5NA00060B-s001
