# Supplementary material for: Microwave-assisted synthesis of self-assembled C-doped-ZnO/g-C3N4 heterojunction catalysts for effective photodegradation of ofloxacin antibiotic
Source: Nanoscale Adv. 2025 Mar 31;7(10):2916–28. doi: 10.1039/d5na00060b (PMC11956032; doi:10.1039/d5na00060b)
Supplement: NA-007-D5NA00060B-s001 [file NA-007-D5NA00060B-s001.pdf]

Supplementary Information (SI) for Nanoscale Advances.
This journal is © The Royal Society of Chemistry 2025

Supplementary document for

Microwave-assisted synthesis of self-assembled C-doped-ZnO/g-C_3N_4 heterojunction catalyst for effective photodegradation of ofloxacin antibiotic

Ha Luu Thi Viet[1], Dao Ngoc Nhiem[2,3,*], Nguyen Van Vinh[3,4], Nguyen Quang-Bac[2], Nguyen Thi Ha Chi[2], Pham Ngoc Chuc[2], Dao Ngoc Hoanh[5], Nguyen Trung Kien[2,3,*]

[1]*Faculty of Chemical Engineering, Industrial University of Ho Chi Minh City, 12 Nguyen Van Bao Street, Ho Chi Minh City 700000, Vietnam*

[2]*Institute of Materials Science, Vietnam Academy of Science and Technology, 18 Hoang Quoc Viet Street, Cau Giay, Hanoi 100000, Vietnam*

[3]*Graduate University of Science and Technology, Vietnam Academy of Science and Technology, 18 Hoang Quoc Viet Street, Cau Giay, Hanoi 100000, Vietnam*

[4]*Joint Vietnam-Russia Tropical Science and Technology Research Center, 63 Nguyen Van Huyen Street, Cau Giay, Hanoi 100000, Vietnam*

[5]*Faculty Of Mechanical Technology, School of Mechanical and Automotive Engineering, Hanoi University of Industry, 298 Cau Dien, Bac Tu Liem, Hanoi 100000, Vietnam*

[*]*Corresponding author:* nhiemdn@ims.vast.ac.vn and nguyentrungkien1009@gmail.com

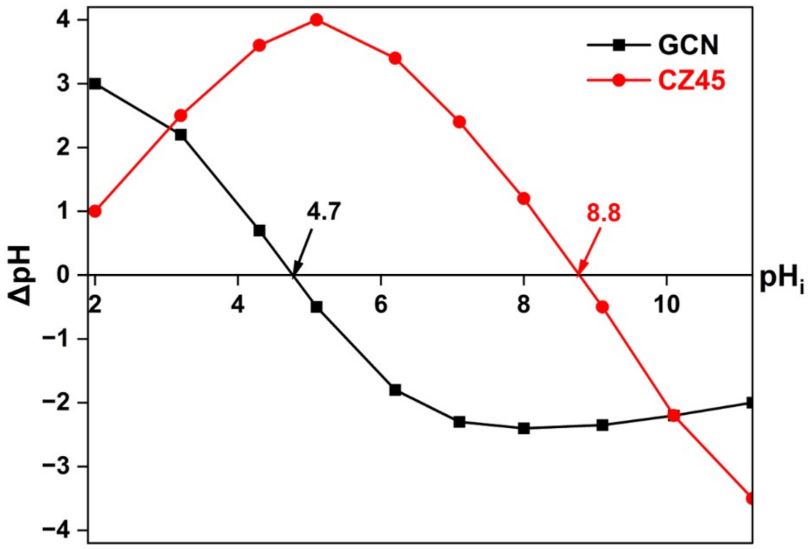

Figure S1. The isoelectric point of GCN and CZ45 samples determined by the drifting method

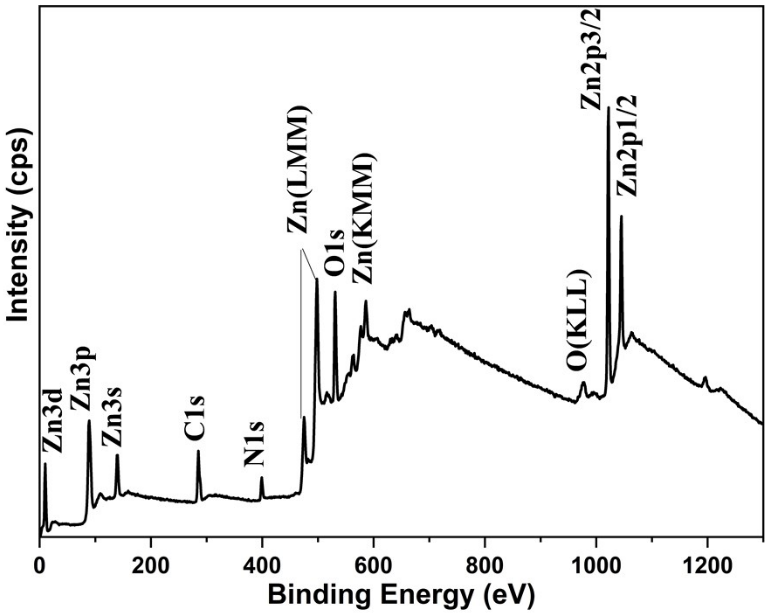

Figure S2. The XPS scan of the CZCN11 sample

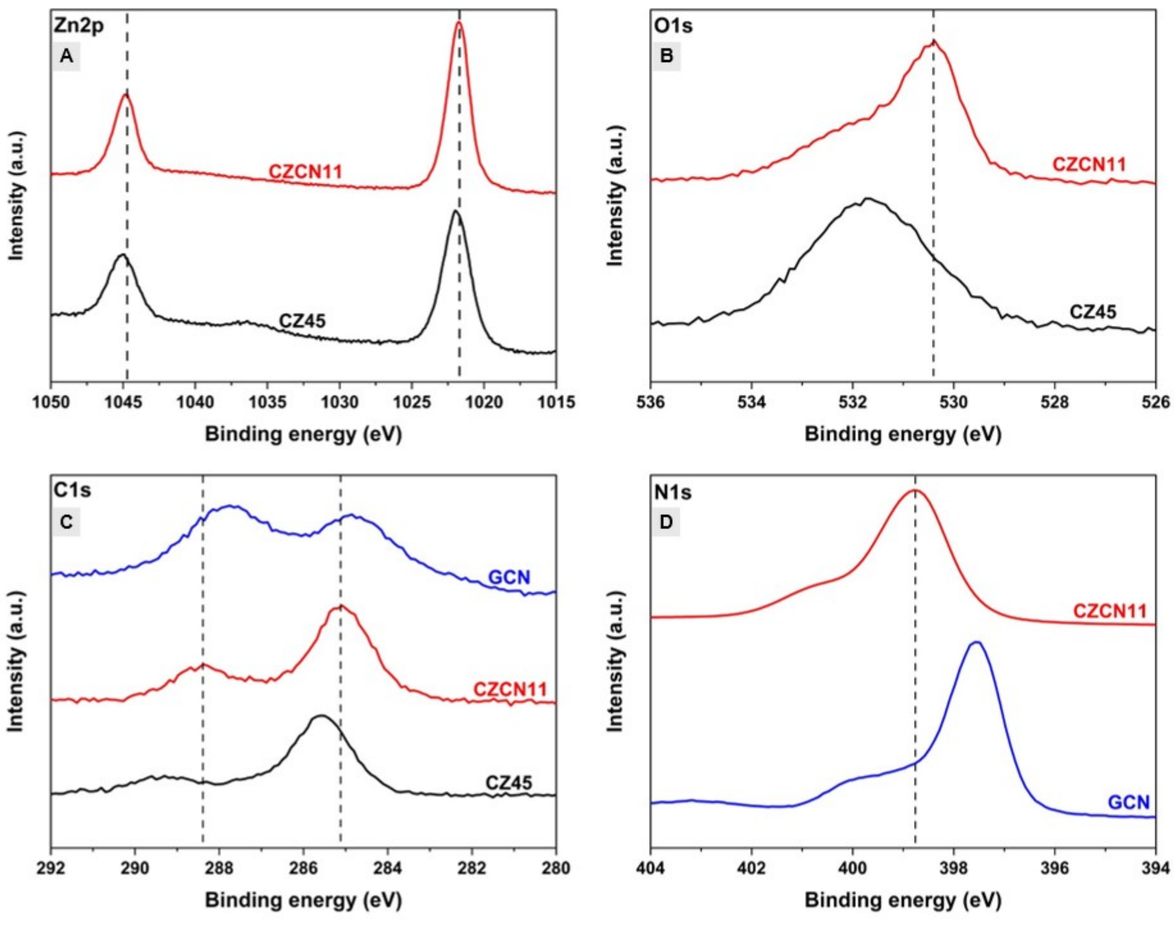

Figure S3. High-resolution XPS spectra of included (A) Zn 2p, (B) O 1s, (C) C 1s, and (D) N 1s in CZ45, GCN, and CZCN materials.

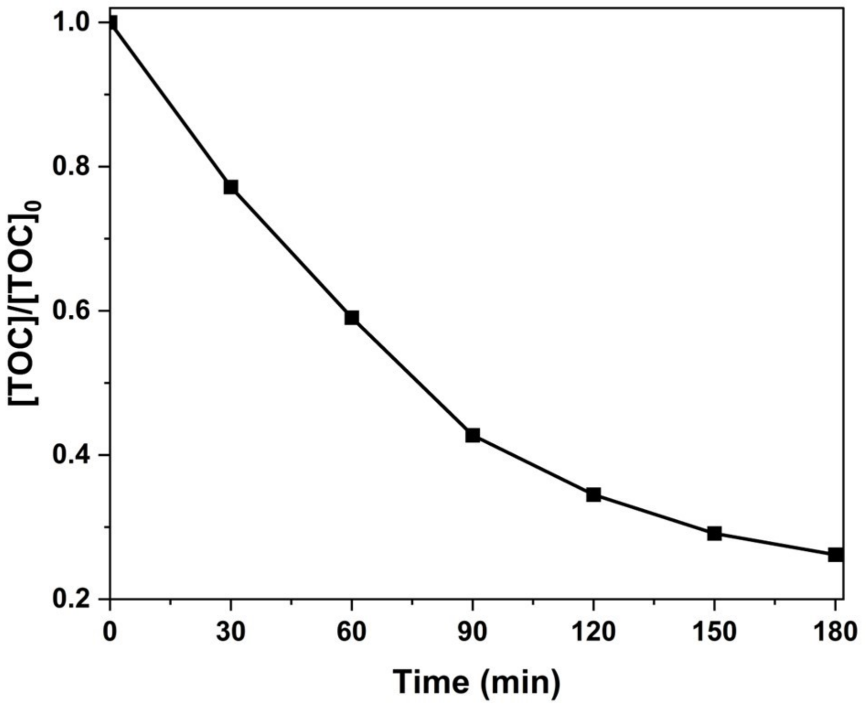

Figure S4. TOC removal during OFL photodegradation using CZCN11 catalyst ([OFL] = 20 ppm, [CZCN11] = 1.0 g/L, pH 7.0)
